# Association of the PROGINS PgR polymorphism with susceptibility to female reproductive cancer: A meta-analysis of 30 studies

**DOI:** 10.1371/journal.pone.0271265

**Published:** 2022-07-15

**Authors:** Chen Zhou, Xiangman Zou, Xiaosha Wen, Zifen Guo

**Affiliations:** 1 The Affiliated Nanhua Hospital, Department of Pharmacy, Hengyang Medical School, Unversity of South China, Hengyang, Hunan, 421001, China; 2 Institute of Pharmacy and Pharmacology, Hunan Province Cooperative Innovation Center for Molecular Target New Drugs Study, Hunan Provincial Key Laboratory of Tumor Microenvironment Responsive Drug Research, University of South China, Hengyang, 421001, China; Fondazione IRCCS Istituto Nazionale dei Tumori, ITALY

## Abstract

**Aims:**

The progesterone response of the nuclear progesterone receptor plays an important role in the female reproductive system. Changes in the function of the progesterone receptor gene may increase the risk of reproductive cancer. The present study performed a meta-analysis to examine whether the progesterone receptor gene *PROGINS* polymorphism was a susceptibility factor for female reproductive cancer.

**Materials and methods:**

We searched the PubMed, Cochrane Library, Web of Science and EMBASE databases for literature on PROGINS polymorphisms and female reproductive cancer published before September 2020. We evaluated the risk using odds ratios [ORs] and 95% confidence intervals via fixed effects models and random-effects models, which were calculated for all five genetic models. We grouped the analyses by race, cancer, and HWE.

**Results:**

Thirty studies comprised of 25405 controls and 19253 female reproductive cancer cases were included in this meta-analysis. We observed that the Alu insertion polymorphism and the V660L polymorphism were significantly associated with female reproductive cancer in the allele and dominant genetic models. The allele genetic model and (Alu-insertion polymorphism: OR = 1.22, 95% CI = 1.02–1.45; V660L polymorphism: OR = 1.02, 95% CI = 1.00–1.13) dominant genetic model (Alu-insertion polymorphism: OR = 1.27, 95% CI = 1.03–1.58; V660L polymorphism: OR = 1.10, 95% CI = 1.011.19) demonstrated a significantly increased risk of female reproductive cancer. A subgroup analysis according to ethnicity found that the Alu insertion was associated with female reproductive cancer incidence in white (Allele model: OR = 1.21, 95% CI = 1.00–1.45; Heterozygous model: OR = 3.44, 95% CI = 1.30–9.09) and Asian (Dominant model: OR = 3.12, 95% CI = 1.25–7.79) populations, but the association disappeared for African and mixed racial groups. However, the V660L polymorphism was significantly associated with female reproductive cancer in the African (Allele model: OR = 2.52, 95% CI = 1.14–5.56; Heterozygous model: OR = 2.83, 95% CI = 1.26–6.35) and mixed racial groups (Dominant model: OR = 1.28, 95% CI = 1.01–1.62). Subgroup analysis by cancer showed that the PROGINS polymorphism increased the risk of cancer in the allele model, dominant mode and heterozygous model, but the confidence interval for this result spanned 1 and was not statistically significant. This sensitivity was verified in studies with HWE greater than 0.5.

**Conclusion:**

Our meta-analysis showed that the progesterone receptor gene Alu insertion and the V660L polymorphism contained in the PROGINS polymorphism were susceptibility factors for female reproductive cancer.

## Introduction

Cancer is a major public health problem worldwide. Cancer is a multifactorial disease, and there is a coordinated relationship between genetic and environmental factors [1,2]. Despite extensive research to prevent cancer, cancer cases continue to increase sharply. Data from the American Cancer Society in 2022 predicts 1.9 million new cancer cases in 2022. More than 609,360 Americans die of cancer annually, which is equivalent to greater than 1,700 people dying of cancer daily [3].

Progesterone is a key regulatory factor in the proliferation and differentiation of female reproductive tract cells. Progesterone inhibits the proliferation of reproductive tract cells by excessive estrogen via the progesterone receptor (PgR) [4–6], and excessive estrogen stimulation increases the risk of female reproductive tract cancer [7]. *PgR* is a member of the nuclear steroid hormone receptor family and is expressed primarily in female reproductive tissues and the central nervous system. It is encoded by a single gene (Gene ID: 5241) located at 11q22–q23 [8], which encodes two isoforms, PgR-B and PgR-A. The two PgR isoforms with different functions come from different transcriptional promoters. PgR-B (114 KDa) is a transcriptional activator and a mediator of cell proliferation, and PgR-A (94 KDa) is a suppressor of transcription. In vitro studies showed that PgR isoforms exhibited different transcriptional regulatory activities. Robert A. et al. [9] found that selective PgR-A knockout induced endometrial epithelial cell proliferation in mice, which suggests that PgR-A is required to control potential adverse reactions of PgR-B. The expression of PgR-A in PgR-B knockout mice was sufficient and necessary to regulate the antiproliferative response of progesterone and estrogen-induced hyperplasia. Prompt changes in the relative expression of these two isoforms or changes in isoform activity or any other genetic mutations may lead to progesterone receptor alienation. The anti-estrogen proliferation effect of progesterone primarily depends on PgR-B, but the excessive expression of PgR-B causes progesterone-dependent proliferation. Progesterone receptor alienation leads to increased susceptibility to female reproductive cancer.

Silencing or mutation of the PgR gene affects the expression of the progesterone receptor. Six variable sites, four polymorphisms, and five common haploids have been detected in the PgR gene. PROGINS contains the Alu insertion in intron 7 of the *PgR* gene, which is completely linked to the unbalanced linkage (LD) with rs1042838 (V660L in exon 4) and rs1042839 (H770H in exon 5) [10]. The alleles of Alu-insertion alter transcript levels and may contribute to disease risk [11]. The PROGINS polymorphism of the human progesterone receptor diminishes the response to progesterone [12]. The PROGINS allele was significantly associated with decreased serum progesterone levels in patients with polycystic ovary syndrome (PCOS) [13].

The current study considered PROGINS as a risk modifier for gynecological benign and malignant diseases, which indicated that PROGINS may affect *PgR* function. Alu insertion of the PROGINS allele was inversely associated with breast cancer risk and ovarian cancer risk in certain races [14–16]. However, only two research reports that concluded that PROGINS affected the risk of endometrial cancer [17,18]. The V660L polymorphism is caused by G > T, which causes a valine > leucine substitution in the fourth exon of the *PgR* gene. No significant association of the PROGINS polymorphism was found in breast or ovarian cancer studies [19,20]. One study on ovarian cancer [21] also failed to find a link, but another study showed that the T allele (leucine) was associated with an increased risk of breast cancer [22]. However, the results of these studies are inconclusive. Therefore, to clarify the role of the PRPGINS PgR polymorphism in female reproductive cancers, we performed a meta-analysis of all eligible case–control studies to derive the overall cancer risk associated with this polymorphism.

## Materials and methods

The current study conformed to the checklist for meta-analysis of genetic association studies specified for the *PLOS One* approach (S1 Table).

### Literature search and identification

This meta-analysis adhered to the PRISMA guidelines. PubMed, Cochrane Library, Web of Science and EMBASE were used to perform a comprehensive search of published related documents. The following search keywords were used: “polymorphism, genetic” or “breast cancer” or “ovarian cancer” or “endometrial cancer” or “gynecologic neoplasm” and “PROGINS” or “V660 L” or “rs1042838” or “rs1042839” or “H770H” or “Z49816.1” or “Alu-insertion”. A search strategy was developed (S2). The last search was updated on September 26, 2020.

### Inclusion and exclusion criteria

The studies were selected using the following criteria.

The following inclusion criteria were used: (a) case-control or cohort study; (b) assessment of PgR polymorphisms for PROGINS and cancer risk; (c) pathology for diagnosis of cancer patients and confirmation that the control was cancer-free; (d) report odds ratios (ORs) and 95% confidence interval (CIs) values or sufficient data to calculate these values; (e) clearly describe genotyping and statistical methods; (f) participants in the control group were in Hardy-Weinberg Balance (HWE); and (g) no language limitations, regardless of sample size.

The following exclusion criteria were used: (a) case reports, comments, comments, and editorial articles; (b) studies of research progress, severity, treatment response, or survival; (c) when overlapping data from the same case series were included in multiple publications, the most recent or most complete study was selected to perform the meta-analysis, and if no information was available, the study was excluded; and (d) literature with specific requirements for the inclusion of cases or. Any differences in the inclusion of the study were resolved via discussion and subsequent consensus.

### Data extraction

Two authors independently extracted the characteristics of the selected study using a standardized protocol, and the third investigator reviewed the results. The following information was extracted from each study: first author, year of publication, study population (country, ethnicity), type of cancer, number of cases and controls, genotype frequencies for cases and controls, and Hardy-Weinberg equilibrium in controls (HWE). We compared key research characteristics, such as location, study time, and authorship, to determine the existence of multiple publications in the same study.

### Quality assessment of the studies

We evaluated the quality based on the NOS quality evaluation to determine the quality of the included literature, and low-quality articles with less than 3 points were excluded. Chen Zhou and Xiangman Zou independently performed the literature search and data extraction. Disputes were discussed and resolved by Xiaosha Wen and Zifen Guo.

### Statistical analysis

STATA software (version 14.0) was used to synthesize the relevant data, and the odds ratio (OR) and 95% confidence interval (CI) were used to evaluate the relationships between PROGINS gene polymorphisms and female reproductive cancer. Five genetic models were used: T2 vs. T1 (allelic), T1T2+T2T2 vs. T1T1 (dominant), T1T2 vs. T1T1 (heterozygous), T2T2 vs. T1T2+T1T1 (recessive), and T2T2 vs. T1T1 (homozygous). Heterogeneity was evaluated using *I*^2^ statistics. When the heterogeneity test found significant heterogeneity (*I*^2^ > 50 or *P* < 0.05), a random-effect model was used. Otherwise, the fixed model was used. When heterogeneity was present in the study, subgroup analysis was performed according to ethnicity, type of disease and HWE of the included cases to examine the sources of heterogeneity. Sensitivity analysis (excluding one study at a time or changing the model) was used to assess the stability of each efficacy index. Begg’s funnel chart and Egger’s test were used to evaluate the publication bias of this study. When *P* < 0.05, publication bias was present in this study.

## Results

### Study selection and characteristics

**Fig 1:**
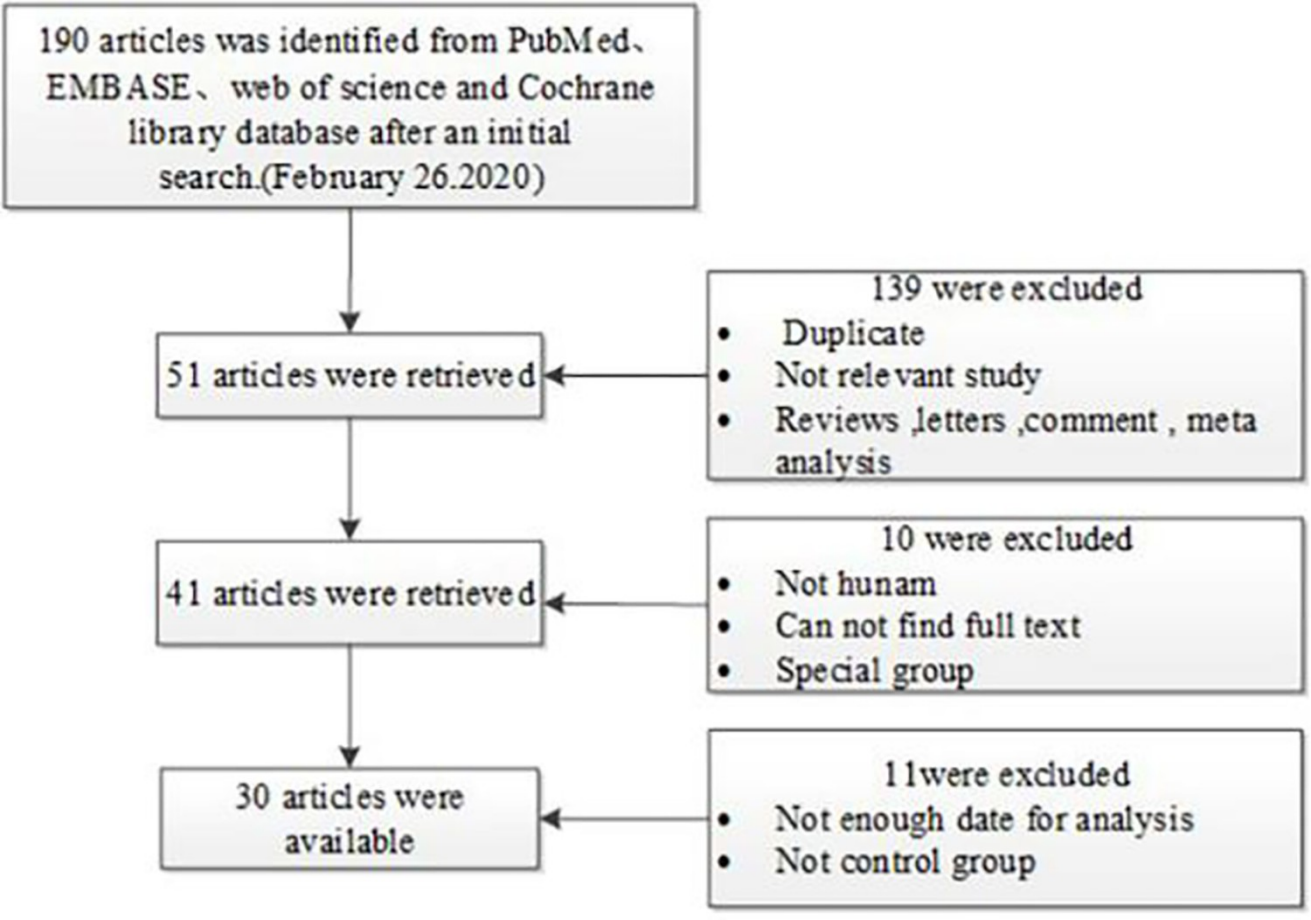
Flowchart showing the meta-analysis literature screening process.

Fig 1 outlines the study selection process in a flowchart following the Preferred Reporting Items for Systematic Reviews and Meta-Analyses (PRISMA) guidelines. A total of 189 articles related to PROGINS polymorphism were retrieved using the retrieval method. Among these articles, 139 articles were excluded after review of the abstracts and unrelated literature, and 11 articles were excluded strictly according to the inclusion and exclusion criteria. Ultimately, 30 articles were included in the meta-analysis (Fig 1) [8,18,19,21–47]. Of the 30 independent studies, 28 studies included genetic frequency analysis of whites, 2 studies included mixed races, 2 studies included Asians and 2 studies included Africans. The types of diseases included breast cancer, ovarian cancer and endometrial cancer. All samples were taken from humans and genotyped using polymerase chain reaction-restriction fragment length polymorphism (PCR–RFLP), DNA sequencing, TaqMan and other genotyping methods (Table 1). The quality of the studies is shown in Table 2.

**Table 1:** Characteristics of the studies included in the meta-analysis.

Study	Country	Ethnicity	Cancer	Detection method	Sample size (case/control)	Genotype frequency						Allele frequency			
						case			controls			Case (%)		Control (%)	
Alu insertion						T_1_T_1_	T_1_T_2_	T_2_T_2_	T_1_T_1_	T_1_T_2_	T_2_T_2_	T_1_	T_2_	T_1_	T_2_
Albalawi, I. A	Saudi Arabia	Asian	BC	PCR–RFLP	100/100	81	18	1	93	6	1	10	90	4	96
Donaldson, C J	USA	white	BC	PCR–RFLP	23/60	17	5	1	41	16	3	84.4	15.2	81.3	18.3
Donaldson, C J -2	USA	African	BC	PCR–RFLP	61/81	56	5	0	73	8	0	95.9	4.1	95.1	4.9
Govindan, S	India	white	BC	PCR–RFLP	157/108	134	23	0	102	6	0	92.7	7.3	97.2	2.8
Junqueira, M. G	Brazil	white	EC	PCR–RFLP	282/121	221	61	0	100	18	3	89.2	10.8	90.1	9.9
Lancaster, J. M -2	USA	white	BC	PCR–RFLP	68/101	55	12	1	79	18	4	89.7	10.3	87.1	12.9
Lancaster, J. M	USA	white	OC	PCR–RFLP	309/397	219	80	10	285	95	17	83.8	16.2	83.8	16.2
Leite, D.B	Brazil	white	OC	PCR–RFLP	80/282	57	12	11	221	61	0	78.8	21.2	89.2	10.8
Manolitsas, T. P	UK	white	BC	PCR–RFLP	292/220	229	61	2	162	54	4	88.9	11.1	85.9	14.1
Manolitsas, T. P -2	UK	white	OC	PCR	231/220	173	52	6	162	54	4	86.1	13.9	85.9	14.1
McKenna, N. J	Ireland	white	OC	S-blot	41/83	26	15	0	58	21	4	81.7	18.3	82.5	17.5
McKenna, N. J-2	Germany	white	OC	S-blot	26/101	17	8	1	88	12	1	80.8	19.2	93.1	6.9
Patricia, G. A	Mexico	white	BC	PCR	481/209	360	103	18	176	33	0	85.6	14.4	92.1	7.9
Runnebaum, I. B	USA	white	OC	PCR	167/496	101	60	6	328	153	15	78.4	21.6	81.6	18.4
Surekha, S	India	white	BC	PCR	250/249	241	7	2	242	7	0	97.8	2.2	98.6	1.4
V660L						GG	GT	TT	GG	GT	TT	G	T	G	T
Gabriel, C. A	USA	white	BC	TaqMan	346/357	236	101	9	255	92	10	82.8	17.2	84.3	15.7
Gabriel, C. A -2	USA	African	BC	TaqMan	86/327	75	11	0	309	16	2	93.6	6.4	96.9	3.1
Clendenen, T	Mix	white	BC	TaqMan	658/1099	846	288	26	1516	523	54	85.5	14.5	84.9	15.1
Fabjani, G	Austria	white	BC	DNA	155/106	119	32	4	78	28	0	87.1	12.9	86.8	13.2
Fernandez, L.P	Spain	white	BC	TaqMan	550/564	354	153	24	375	154	15	81.1	18.9	83.1	16.9
Johnatty, S.E	Australia	white	BC	PCR–RFLP	1444/583	1017	380	47	409	160	14	83.6	16.4	83.9	16.1
Lee, E	USA	MIX	EC	TaqMan	198/1077	170	25	3	954	114	9	92.2	7.8	93.9	6.1
Lee, E -2	USA	white	EC	TaqMan	379/836	259	109	11	615	199	22	86	14	90.2	9.8
Lundin, E	MIX	white	EC	TaqMan	391/705	281	96	14	540	147	18	84.1	15.9	87	13
O’Mara, T. A	Singapore	Asian	EC	TaqMan	528/1538	414	151	17	1147	361	30	84.1	15.9	86.3	13.7
O’Mara, T. A -2	UK	white	EC	TaqMan	1086/1591	765	294	27	1123	434	34	84	16	84.2	15.8
O’Mara, T. A -3	Australia	white	EC	TaqMan	1220/1354	867	323	30	933	383	38	84.3	15.7	83.1	16.9
Pearce, C. L	USA	white	OC	DNA	267/397	173	82	12	279	111	6	80.1	19.9	84.5	15.5
Pearce, C. L-2	USA	white	BC	DNA	1715/2505	1400	252	15	2025	363	37	91.5	8.5	91	9
Pooley, K. A	Englishman	white	BC	TaqMan	2345/2281	1302	517	42	1461	513	39	83.9	16.1	85.3	14.7
Romano, A	Netherlands	white	BC	PCR–RFLP	167/31	123	41	3	22	7	2	85.9	14.1	82.3	17.7
Romano, A	German	white	BC	PCR–RFLP	545/443	399	133	14	347	87	9	85.4	14.6	88.1	11.9
Romano, A -2	German	white	OC	PCR–RFLP	67/443	42	24	1	347	87	9	80.6	19.4	88.1	11.9
Spurdle, A. B	Australia	white	OC	PCR–RFLP	551/298	395	144	12	203	90	5	84.8	15.2	83.2	16.8
Terry, K. L	USA	white	OC	TaqMan	895/939	648	223	25	612	298	29	84.9	15.1	81	19
De vivo, I	USA	white	BC	TaqMan	1252/1660	869	348	35	1186	434	40	83.3	16.7	84.5	15.5
Tong, D	Austrian	white	OC	DNA	226/194	167	50	9	141	52	1	85	15	86.1	13.9
Quaye, L	UK/USA	white	OC	TaqMan	1424/2408	1005	377	42	1819	526	63	83.8	16.2	86.5	13.5
Ghali, R. M	Tunisia	white	BC	TaqMan	183/216	127	50	6	172	37	7	83.1	16.9	88.2	11.8

PCC, population-based case–control study, HCC, hospital-based case–control study, PCR–RFLP PCR-restriction fragment length polymorphism, BC, Breast cancer, EC, Endometrial cancer, OC, Ovarian cancer, DNA, DNA sequencing, S-blot, Southern blot.

**Table 2:** Article quality evaluation.

Study	Adequate case definition	Definition of controls	Comparability	HWE>0.05	PCC	PMH (Past Medical History)	Unified detection method	Article quality
Alu insertion								
Albalawi 2020 [47]	1	1	1	0	0	1	1	5
Donaldson 2002 [28]	1	1	1	1	0	0	1	5
Govindan, S 2007 [34]	1	1	1	1	0	0	1	5
Junqueira, M.G 2007 [18]	1	1	1	1	0	1	1	6
Lancaster 1998 [25]	1	0	0	0	0	0	1	2
Lancaster, J.M 2003 [30]	1	1	1	0	1	1	1	6
Leite, D.B. 2008 [37]	1	1	1	0	0	1	1	5
Manolitsas TP 1997 [24]	1	0	1	1	1	0	1	5
McKenna 1995 [23]	0	0	1	1	0	0	1	3
Patricia Gallegos-Arreola, M 2015 [15]	1	1	1	1	1	1	1	7
Runnebaum, I.B 2001 [26]	1	1	1	1	1	1	1	7
Surekha 2009 [39]	1	1	1	1	1	1	1	7
V660L								
Gabriel, C. A.2013 [44]	1	1	1	1	0	1	1	6
Clendenen, T 2013 [43]	1	1	1	1	1	1	1	7
Fabjani, G 2002 [29]	1	1	1	1	1	1	1	7
Fernandez, L.P 2006 [31]	1	1	1	1	0	1	1	6
Johnatty, S.E 2008 [36]	1	1	1	1	1	1	1	7
Lee, E 2010 [40]	1	1	1	0	1	1	1	6
Lundin, E 2012 [42]	1	1	1	0	1	1	1	6
O’Mara, T.A 2011 [41]	1	1	1	1	1	1	1	7
Pearce, C.L. 2005 [22]	1	1	1	1	1	0	1	6
Pooley, K.A 2006 [32]	1	1	1	1	1	0	1	6
Romano A 2007 [12]	1	1	1	1	1	1	1	7
Romano, A 2006 [33]	1	1	1	1	1	0	1	6
Spurdle 2001 [21]	1	1	1	1	1	1	1	7
Terry, K.L. 2005 [8]	1	1	1	1	1	1	1	7
De vivo 2004 [19]	1	1	1	1	1	0	1	6
Tong, D. 2001 [27]	1	1	1	1	1	0	1	6
Quaye 2009 [38]	1	1	1	0	1	1	1	6
Ghali RM 2020 [46]	1	1	1	0	0	1	1	5

low quality, <3; Medium quality,3–4; high quality, ≥5.

### Alu-insertion polymorphism and the risk of female reproductive cancer

**Fig 2:**
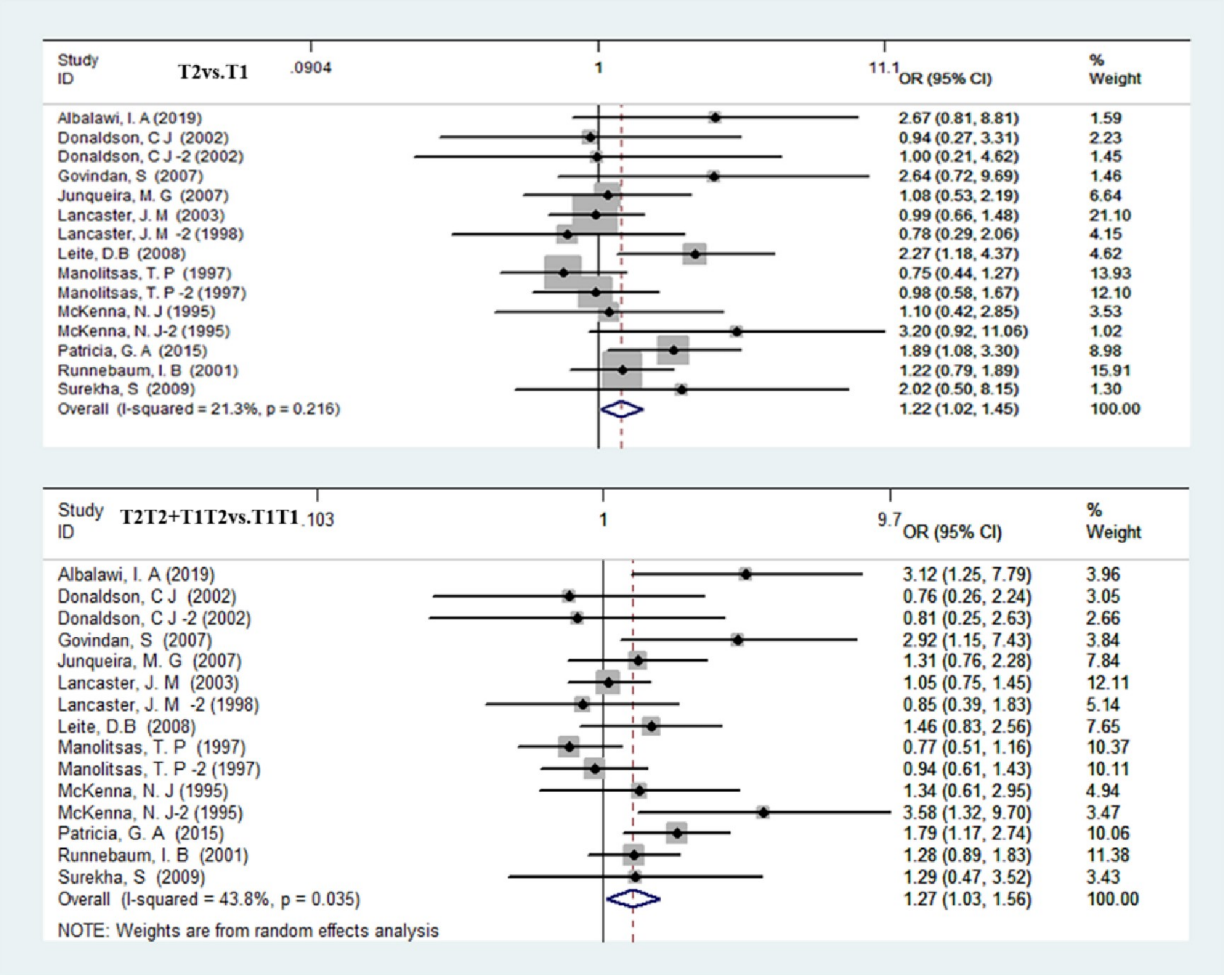
Forest plot of overall cancer risk associated with Alu-insertion PgR polymorphism (T2 vs. T1 and T2T2+T1T2vs.T1T1).

**Fig 3:**
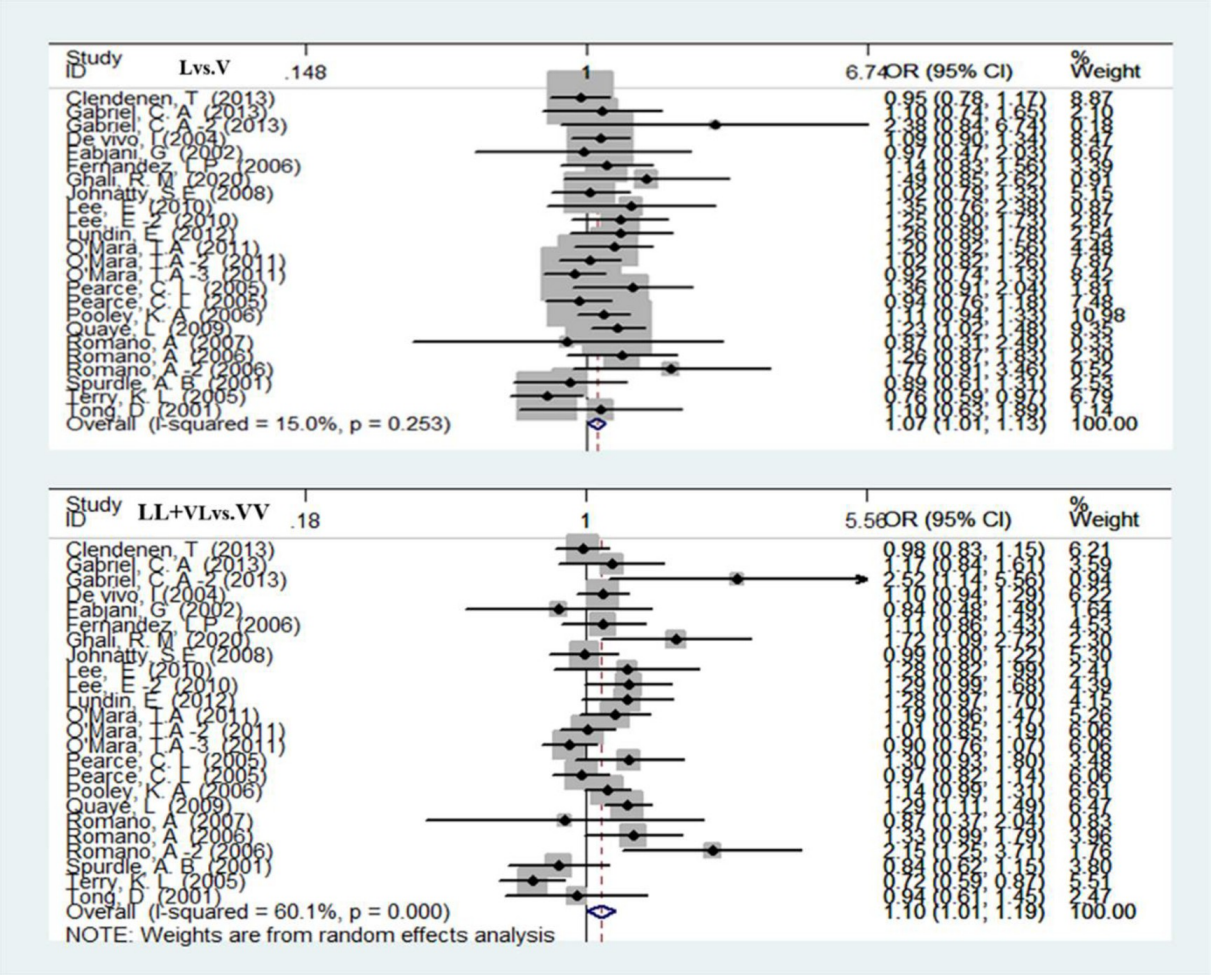
Forest plot of overall cancer risk associated with V660L PgR polymorphism (L vs. V and LL+VL vs. VV).

We counted the Alu-insertion locus and the susceptibility to female reproductive cancer in the five models of allele genetic model (T2 vs. T1), homozygous genetic model (T2T2 vs. T1T2), heterozygous genetic model (T1T2 vs. T1T1), dominant genetic model (T1T2+T2T2 vs. T1T1) and recessive genetic models (T2T2 vs. T1T2+T1T1) (Table 3). The meta-analysis showed a significant association between Alu-insertion polymorphisms and the risk of female reproductive cancer in the allele genetic model (OR = 1.22 95% CI = 1.02–1.45), the dominant genetic model (OR = 1.27 95% CI = 1.03–1.58), and the heterozygote genetic model (OR = 1.19 95% CI = 1.03–1.38) (Figs 2 and 3). A significant association was found in the allele genetic model of the white group (OR = 1.21 95% CI = 1.00–1.45) (Table 4).

**Table 3:** Meta-analysis of the association between the PROGINS polymorphism and female reproductive cancer susceptibility.

Polymorphism	Genetic model	Case/Control	Test of association		Heterogeneity			Publication bias	
			OR(95%CI)	*P*	I^2^(%)	*P*Het	Model	Egger‘s test p value	Begg’s test p value
Alu insertion	T2 vs. T1	2568/2828	1.22[1.02,1.45]	0.027 *	21.3	0.218	F	0.373	0.168
	T2T2+T1T2 vs.T1T1	2568/2828	1.27[1.03,1.58]	0.023*	43	0.035	R	0.373	0.201
	T2T2 vs.T1T2+T1T1	2568/2828	1.18[0.55,2.55]	0.670	52.1	0.015	R	0.360	0.469
	T2T2 vs.T1T1	2046/2322	1.23[0.57,2.65]	0.605	52.2	0.014	R	0.428	0.455
	T1T2 vs.T1T1	2509/2772	1.19[1.03,1.38]	0.019*	39	0.058	F	0.428	0.263
V660L	L vs. V	16685/22577	1.07[1.00,1.13]	0.031*	15	0.253	F	0.130	0.081
	LL+VL vs. VV	16685/22577	1.10[1.01,1.19]	0.027*	60.1	0.000	R	0.503	0.265
	LL vs. VL+VV	16685/22577	1.13[0.99,1.29]	0.075	0	0.476	F	0.413	0.244
	LL vs. VV	12481/17361	1.07[0.93,1.23]	0.325	5	0.392	F	0.673	0.219
	VL vs. VV	16257/22084	1.09[1.00,1.18]	0.056	60.7	0.000	R	0.385	0.204

F: Fixed model, R: Random model.

**Table 4:** Pooled odds ratios (ORs) in subgroups.

SNP/subgroups	No. of study	Allele model			Dominant model			Recessive model			Homozygous model			Heterozygous model		
		OR	95%CI	*P*	OR	95%CI	*P*	OR	95%CI	*P*	OR	95%CI	*P*	OR	95%CI	*P*
Alu insertion																
OC	5	1.20	0.95–1.51	0.125	1.23	0.96–1.56	0.100	1.59	0.56–4.49	0.382	1.67	0.60–4.69	0.330	1.13	0.93–1.37	0.232
BC	7	1.28	0.95–1.72	0.101	1.30	0.86–1.97	0.219	1.14	0.32–4.04	0.836	1.17	0.33–4.29	0.818	1.23	0.97–1.55	0.086
EC	1	1.08	0.53–2.19	0.828	1.31	0.76–2.28	0.170	0.06	0.00–1.17	0.063	0.06	0.00–1.27	0.071	1.53	0.86–2.73	0.146
white	9	1.21	1.00–1.45	0.046*	1.23	0.99–1.54	0.066	1.40	0.62–3.14	0.413	1.44	0.84–3.28	0.377	1.14	0.98–1.33	0.099
Asian	1	2.67	0.81–8.81	0.108	3.12	1.25–7.79	0.015*	1.00	0.06–16.21	1	1.15	0.07–18.65	0.923	3.44	1.30–9.09	0.013*
African	1	1.00	0.21–4.62	0.996	0.81	0.25–2.63	0.731	——	——	——	——	——	——	0.81	0.25–2.63	0.731
Mix	1	1.08	0.53–2.19	0.828	1.31	0.75–2.28	0.329	0.06	0.00–1.17	0.063	0.06	0.00–1.27	0.071	1.53	0.86–2.73	0.019
HWE > 0.05	8	1.21	0.98–1.50	0.076	1.27	0.98–1.65	0.066	0.85	0.38–1.91	0.043	1.12	0.48–2.61	0.797	1.22	1.02–1.45	0.026
HWE < 0.05	4	1.23	0.90–1.68	0.188	1.30	0.85–1.98	0.230	1.85	0.22–15.66	0.004	1.88	0.23–15.50	0.556	1.13	0.86–1.48	0.379
V660L																
OC	6	1.06	0.94–1.20	0.334	1.09	0.82–1.45	0.566	1.24	0.94–1.64	0.126	1.19	0.89–1.59	0.230	1.05	0.78–1.43	0.733
BC	10	1.06	0.98–1.15	0.139	1.09	1.00–1.19	0.052	1.07	0.88–1.29	0.518	1.00	0.82–1.22	0.979	1.09	0.99–1.20	0.064
EC	3	1.07	0.96–1.20	0.215	1.10	0.97–1.26	0.137	1.16	0.90–1.50	0.256	1.11	0.85–1.44	0.461	1.09	0.96–1.23	0.195
white	17	1.06	0.99–1.12	0.081	1.07	0.98–1.17	0.134	1.10	0.95–1.27	0.189	1.06	0.91–1.22	0.471	1.06	0.97–1.16	0.209
Asian	1	1.20	0.92–1.56	0.186	1.19	0.96–1.47	0.109	1.51	0.83–2.76	0.179	1.35	0.73–2.53	0.341	1.16	0.93–1.45	0.190
African	1	2.38	0.84–6.74	0.103	2.52	1.14–5.56	0.022*	0.75	0.04–15.82	0.855	0.29	0.02–6.55	0.434	2.83	1.26–6.35	0.012*
Mix	2	1.35	0.76–2.38	0.305	1.28	1.01–1.62	0.041*	1.49	0.80–2.79	0.212	1.23	0.65–2.42	0.499	1.25	0.97–1.60	0.081
HWE > 0.05	15	1.04	0.97–1.11	0.229	1.03	0.93–1.14	0.003	1.17	1.00–1.37	0.46	1.15	0.98–1.35	0.084	1.03	0.95–1.13	0.452
HWE < 0.05	6	1.16	1.02–1.31	0.020	1.34	1.10–1.64	0.003	1.01	0.76–1.29	0.975	0.85	0.64–1.14	0.277	1.29	1.06–1.57	0.009

### Val 660 Leu polymorphism and the risk of female reproductive cancer

A total of 16685 cancer patients and 22577 healthy women in 18 studies were used to assess the relationship between the V660 locus and female reproductive cancer risk using the allele genetic model (L vs. V), homozygous genetic model (LL vs. VV), heterozygous genetic model (VL vs. VV), dominant genetic model (VL+LL vs. VV) and recessive genetic models (LL vs. VL+VV). The V660L mutation increased the risk of female reproductive cancer in the allele genetic model (OR = 1.02 95% CI = 1.00–1.13) and dominant genetic model (OR = 1.10 95% CI = 1.01–1.19). The heterozygote genetic model confirmed (OR = 1.09 95% CI = 1.00–1.18) that the V660L mutation increased the risk of female reproductive cancer (Fig 3 and Table 3).

The subgroup analysis found a significant association under the dominant genetic model (OR = 1.21 95% CI = 1.00–1.45) of the breast cancer group (Table 4).

### Publication bias

Begg’s and Egger’s analyses showed that no publication bias in the Alu insertion or V660L (Table 3).

### Sensitivity and heterogeneity

A sensitivity analysis was performed to determine whether changes in the inclusion criteria for meta-analysis affected the final results. The author deleted individual studies involved in each meta-analysis to reflect the impact of a single dataset on the merged ORs. Most of the corresponding merged ORs did not change substantially (data not shown). We also changed the effect model to test the impact on the results, and no substantial changes were found on the combined OR, which showed that our results were statistically robust. I^2^ statistics were used to test the heterogeneity (Table 3), and no heterogeneity was observed in any of the genetic models.

## Discussion

Current evidence indicates that progesterone plays a vital role in regulating female reproduction. The physiological role of progesterone is mediated by the progesterone receptor (PgR), which includes a total of 8 exons and 7 introns [48]. PgR-A and PgR-B are the two subtypes of PgR. The co-expression levels in most normal progesterone-targeted cells are similar. The balance between subtypes regulates the expression of many other genes. Abnormal expression of PgR-A or PgR-B causes a significant change in the ratio between subtypes, which leads to changes in the transmission of progesterone information, and these changes affect physiological functions and trigger a series of serious physiological consequences. The Alu insertion together with V660L and H770H is called PROGINS, which is an important polymorphism of the *PgR* gene. The Alu insertion affects the binding properties of receptors and hormones and induces amino acid changes, which cause female reproductive cancer.

Our meta-analysis included 30 studies with 25405 controls and 19253 female reproductive cancer cases. These studies examined the relationship between *PgR* gene PROGINS polymorphisms (Alu insertion and V660L) and female reproductive tract cancer. Our meta-analysis results demonstrated a significant association between PROGINS and female reproductive cancer, and PROGINS mutations increased the risk of female reproductive cancer. We also performed a sensitivity analysis to test the validity of the results, and the results of the meta-analysis were stable. The association between *PgR* mutations and female reproductive cancer varies between races. The meta-analysis of the dominant genetic model of the Alu-insertion polymorphism showed that women with T2 mutations had a significantly higher risk of developing female reproductive tract cancer than women with T1T1 genotypes in the general population. There was a significant association between Alu insertion and female reproductive cancer in whites (OR = 1.25, 95% CI = 1.01–1.56), but this association disappeared in Asians and Africans. The difference in correlation may be caused by several factors. First, the frequency of Alu insertion is different due to different ethnic groups, different ethnic groups of genetic backgrounds, different lifestyles, and different environmental factors. Second, there are few reports of the locus in Asians and Africans.

Although some studies showed linkage disequilibrium reactions between Alu insertions and V660L, V660L cannot replace Alu insertions in the analysis of genetic polymorphisms based on these meta-analysis data. The disease correlation between the two polymorphisms was different between ethnicities. Alu insertion was associated with female reproductive cancer incidence in white (Allele model: OR = 1.21, 95% CI = 1.00–1.45; Heterozygous model: OR = 3.44, 95% CI = 1.30–9.09) and Asian populations (Dominant model: OR = 3.12, 95% CI = 1.25–7.79), but the association disappeared for African and mixed racial groups.

Our results showed a significant relationship between V660L and the susceptibility to female reproductive cancer in the allele genetic model, dominant genetic model and heterozygous genetic model.

However, our research has some potential limitations. First, studies that met the inclusion criteria or were unpublished may have been missed. Second, although the control group was primarily selected from healthy people, some people did not mention their physiological status or whether they had benign disease. Finally, 26 studies included whites in the ethnic subgroup analysis, but few studies included Asians and Africans. Therefore, the differences in the associations between different ethnic subgroups should be carefully interpreted. In conclusion, although there are limitations, the results in this article provide significant evidence that PROGINS increases the risk of female reproductive cancer.

## Supporting information

S1 Checklist (DOCX)

S1 Table (XLSX)
