# Supplementary material for: Association of the PROGINS PgR polymorphism with susceptibility to female reproductive cancer: A meta-analysis of 30 studies
Source: PLoS One. 2022 Jul 15;17(7):e0271265. doi: 10.1371/journal.pone.0271265 (PMC9286292; doi:10.1371/journal.pone.0271265)
Supplement: S1 Checklist — (DOCX) [file pone.0271265.s001.docx]

**Meta-analysis on Genetic Association Studies Checklist | PLOS ONE**

|  | Item | Section name and paragraph number within manuscript |
| --- | --- | --- |
|  | **Introduction** |  |
| 1 | Provide a detailed justification for the polymorphism studied; if a single polymorphism was analyzed, give details as to why others were not included in the meta-analysis. | 2-4 |
| 2 | Provide a detailed justification for the population(s) and clinical condition studied. | 1-4 |
|  | **Methods** |  |
| 3 | Provide full details of the search strategy employed; outline the full electronic search strategy –specific combination of keywords and any limits applied- for at least one database. Specify whether synonyms of polymorphisms/genes (e.g. SNP number) were searched. | Database searched File; Literature search and Identification |
| 4 | Report full details on the inclusion and exclusion criteria applied for selecting studies.  *Please list the excluded articles and the reasons for exclusion of each article in a supplementary file.* | Inclusion and exclusion criteria |
| 5 | Provide details on how the quality of the studies included in the analyses was assessed. | Quality Assessment of the studies  Table 2  Statistical analysis 1 |
| 6 | Describe steps taken to contact study authors to identify additional studies and to request missing data. | Data extraction 1 |
| 7 | Describe how environmental effects were adjusted for, if this adjustment was not conducted, outline the reasons for this. | Not included in the primary studies. |
| 8 | Describe the methods of handling heterogeneity/between-study variance. | Statistical analysis  1 |
| 9 | Describe how the Hardy-Weinberg equilibrium and linkage disequilibrium were assessed. | Statistical analysis  1 |
| 10 | Describe and justify the choice of model for the analyses (per-allele vs per-genotype vs genetic model-free, random effects vs fixed effects). | Statistical analysis 1 |
| 11 | Describe whether a sensitivity analysis has been completed. | Statistical analysis 1 |
| 12 | Describe whether an assessment of the effects of population stratification has been conducted. | Statistical analysis 1 |
| 13 | Describe whether study-specific results have been assessed and if so the reasons for this (e.g. forest plot). | Statistical analysis 1 |
|  | **Results** |  |
| 14 | Include flow diagram for the studies included in the meta-analysis as the first figure for the manuscript | study selection and characteristics  Figure 1 |
| 15 | Report details on allele/genotype prevalence. | Meta-analysis Databases: association between PROGINS polymorphism and  female reproductive cancer  1  Table 1 |
| 16 | Report the effect size estimates and p values for each analysis. | Meta-analysis Databases: association between PROGINS polymorphism and  female reproductive cancer  Figure 2, 3 and table3, 4 |
|  | **Discussion** |  |
| 17 | Discuss the limitations of the meta-analysis, including genotyping errors/bias and publication bias. | 3-5 |
| 18 | If the meta-analysis identifies an association within a subgroup of the population studied but not another, discuss the implications of these results, and if applicable the possibility of subgroup-specific publication bias. | 2 |
| 19 | Discuss the suitability of the sample size employed to the research question and the power of the study. | 5 |
