# Supplementary material for: Association of the PROGINS PgR polymorphism with susceptibility to female reproductive cancer: A meta-analysis of 30 studies
Source: PLoS One. 2022 Jul 15;17(7):e0271265. doi: 10.1371/journal.pone.0271265 (PMC9286292; doi:10.1371/journal.pone.0271265)
Supplement: Attachment — Submitted filename: Comments PLOS ONE.doc [file pone.0271265.s003.doc]

Detailed comments for the author's consideration

Review of the paper by *Chen Zhou, Xiangman Zou, Xiaosha Wen， Zifen Guo entitled: “Association of PROGINS PgR Polymorphism with susceptibility to female reproductive cancer: A metaanalysis based on 30 studies” The work presented is interesting in terms of demonstrating the association of PROGINS PgR Polymorphism with susceptibility to female reproductive cancer. The manuscript is well written and results are well described with proper statistics. The language is appropriate, technical and easy to read. However, I have some minor recommendations

**Comment 1:** The highlights of the manuscript should be written properly with more conclusive meaning.

**Comment 2:** The first sentence of the abstract is not properly written. It should be rewritten with proper meaning.

**Comment 3:** The first para of introduction section has 2020 data. Update the para with 2021/22 epidemological and incidence data.

**Comment 4:** In second para of introduction section there are some sentences related to estrogen which creates confusion. Remove the estrogen related sentences or If not rewrite the sentence with proper meaning. Additionally, in second para “vitro” should be replaced with “invitro”

**Comment 5:** Add some lines about PROGINS and its functions in para 3 of introduction section.

**Comment 6**: There are various spelling and grammatical mistakes such as “conformed” in materials and methods section. Kindly go through the whole manuscript to edit grammatical mistakes.

**Comment 7:** Manuscript contains old and outdated references, replace them with latest and updated references wherever possible.
