# Supplementary material for: Association of the PROGINS PgR polymorphism with susceptibility to female reproductive cancer: A meta-analysis of 30 studies
Source: PLoS One. 2022 Jul 15;17(7):e0271265. doi: 10.1371/journal.pone.0271265 (PMC9286292; doi:10.1371/journal.pone.0271265)
Supplement: Attachment — Submitted filename: Response to Reviewers.doc [file pone.0271265.s004.doc]

Dear the editor and reviewers:

Thank you very much for giving us an opportunity to revise our manuscript **(PONE-D-21-14829)** titled **Association of PROGINS PgR Polymorphism with susceptibility to female reproductive cancer: A meta-analysis based on 30 studies**. We appreciate the positive and constructive comments and suggestions from the editor and reviewers, which significantly improved the quality of our manuscript. We have carefully revised the manuscript accordingly with all changes shown in red in the paper. Please see the detailed responses below.

**Reviewers 1' Comments to Author**

This study is a meta-analysis of the association between PgR gene polymorphisms and female reproductive cancer. This is a very interesting study, but I would like to point out the following points.

**Comment 1:** Most of the papers that authors adopted for this meta-analysis are on cancers that occur in specific organs (breast, endometrial, and ovarian cancer) and gene polymorphisms in PgR. Does the role of PgR play a similar role in each of these organs? Is it justified to perform a meta-analysis together with female reproductive cancer without considering organ specificity? For example, past epidemiological studies have shown that hormone replacement therapy, including progesterone, has different effects on breast and endometrial cancer risks.

**RESPONSE:** Thank you very much for your constructive suggestions. Progesterone and progesterone receptors (PgR) are essential for the development and cyclical regulation of hormone-responsive tissues including the breast and reproductive tract. We think it is reasonable to conduct a Meta-analysis on female reproductive cancers. Human is an organic whole, and the regulation of genes by genetic polymorphisms is holistic. As a steroid hormone, the effects of progesterone exerts on the female reproductive system are complicated.

The main subtypes of PgR are PgR-A and PgR-B mediating the main progesterone responses, but the roles of PgR-A and Pgr-B in different organs are not the same. PgR-A is essential for ovarian normal function in ovarian cancer, while its expression is decreased or lost in ovarian cancer. PgR-B has the functions of anti-proliferation (such as anti-aging and anti-apoptosis), playing a dominant role in ovarian cancer. In breast cancer, PgR has been shown to rapidly activate the Src/p21Ras/Erk, PI3K/Akt, and JAK/STAT pathways that contribute to the proliferative effect of progesterone in breast cancer cells, but unliganded PgR has been shown to suppress growth and inflammatory responses in breast cancer cells. The PgR status of endometrial tumors has been controversial. One of the previous studies showed the predominance of PgR-B in advanced endometrial tumors, but another study noted the loss of both subtypes in advanced endometrial cancers; additionally, a third study showed that only PgR-A was expressed in poorly differentiated endometrial cancer cell lines. At present, the imbalance between the expression of PgR-A and PgR-B is one of the pathogenesis of endometrial cancer.

**Comment 2：**From the viewpoint of Comment 1, the interpretation of the results of this study, which examined the relationship between female reproductive cancer and gene polymorphisms in PgR, is difficult to interpret and seems to have low clinical significance. I think it is more clinically significant to focus on organ-specific meta-analysis and discuss whether there are any differences.

**RESPONSE:** Thank you very much for the good advice. As to the data presented in this paper, the risk propensity for female reproductive cancers is the same as that for ovarian, breast, and endometrial cancers. The data in table 4 suggest that PROGINS gene polymorphisms may be associated with a slightly increased risk of ovarian, breast, and endometrial cancers.

**Comment 3：** Abstract，In the Material and Method section, it is said that the analysis was performed for the race, cancer, and HWE groups, but the in section of Result, the description of the analysis result is only for race.

**RESPONSE:** Thank you very much for the good advice. We added the analysis results of cancer and HWE groups to the Abstract.

Abstract： Subgroup analysis by cancer, PROGINS polymorphism increase the risk of cancer in the Allele model, Dominant mode and Heterozygous model, but the confidence interval for this result spanned 1 and was not statistically significant. At the same time, this sensitivity was also verified in studies with HWE greater than 0.5.

**Comment 4：** Figure 1，Check if the numbers in the study consort are correct.　 The total number of excluded studies and the number of non-excluded studies do not match.

**RESPONSE:** Thank you very much for the good advice. We verified the data in Figure 1 and corrected this error.

Fig 1. Flowchart showing the meta-analysis literature screening process

**Reviewers 2' Comments to Author:**

The work presented is interesting in terms of demonstrating the association of PROGINS PgR Polymorphism with susceptibility to female reproductive cancer. The manuscript is well written and results are well described with proper statistics. The language is appropriate, technical and easy to read. However, I have some minor recommendations.

**Comment 1:** The highlights of the manuscript should be written properly with more conclusive meaning.

**RESPONSE:** Thank you very much for the good advice. We have refined the highlights.

Highlights

Alu insertion may be a susceptible factor for reproductive cancer in white and Asian populations, and the V660L polymorphism may be a susceptible factor for female reproductive cancer in African populations.

**Comment 2:** The first sentence of the abstract is not properly written. It should be rewritten with proper meaning.

**RESPONSE:** Thank you very much for the good advice. We rewrote this sentence.

Aims: The progesterone response of the nuclear progesterone receptor plays an important role in the female reproductive system.

**Comment 3:** The first para of introduction section has 2020 data. Update the para with 2021/22 epidemological and incidence data.

**RESPONSE:** Thank you very much for the good advice. We have updated the latest epidemiological data for 2022.

Despite extensive research to prevent cancer, cancer cases continue to increase sharply. Data from the American Cancer Society in 2022 predicts 1.9 million new cancer cases in 2022. More than 609,360 Americans die of cancer annually, which is equivalent to greater than 1,700 people dying of cancer daily [3].

**Comment 4:** In second para of introduction section there are some sentences related to estrogen which creates confusion. Remove the estrogen related sentences or If not rewrite the sentence with proper meaning. Additionally, in second para “vitro” should be replaced with “invitro”.

**RESPONSE:** Thank you very much for the good advice. We rewrote the sentence to clarify it and replaced the word.

Progesterone inhibits the proliferation of reproductive tract cells by excessive estrogen via the progesterone receptor (PgR) [4-6], and excessive estrogen stimulation increases the risk of female reproductive tract cancer [7].

**Comment 5:** Add some lines about PROGINS and its functions in para 3 of introduction section.

**RESPONSE:** Thank you very much for the good advice. We added the functions of PROGINS to the Introduction.

The PROGINS polymorphism of the human progesterone receptor diminishes the response to progesterone [12]. The PROGINS allele was significantly associated with decreased serum progesterone levels in patients with polycystic ovary syndrome (PCOS) [13].

**Comment 6**: There are various spelling and grammatical mistakes such as “conformed” in materials and methods section. Kindly go through the whole manuscript to edit grammatical mistakes.

**RESPONSE:** Thank you very much for the good advice. We carefully revised the sentences in the article, and we have updated and revised our manuscript through the language editing service from AJE (Verification Code: 0283-9E24-1846-6C24-A673). Certificate of language editing as follows:

**Comment 7:** Manuscript contains old and outdated references, replace them with latest and updated references wherever possible.

**RESPONSE:** Thank you very much for the good advice. We updated the current references, but the references the data extracted from could not be replaced.
